# The Extent of Lymphadenectomy at the Time of Radical Cystectomy for Bladder Cancer and its Impact on Prognosis and Survival

**DOI:** 10.1100/tsw.2005.115

**Published:** 2005-11-11

**Authors:** David Y. Josephson, John P. Stein

**Affiliations:** Department of Urology, Norris Comprehensive Cancer Center, University of Southern California Keck School of Medicine, Los Angeles, California, USA

**Keywords:** bladder cancer, cystectomy, lymphadenectomy, lymph node metastases

## Abstract

Radical cystectomy has become a standard and effective treatment for muscle-invasive bladder cancer, however, the role and appropriate extent of a concomitant lymphadenectomy continues to evolve. We performed a detailed review of the English medical literature pertaining to the historical development and rationale for an extended lymphadenectomy in patients undergoing radical cystectomy. An historical perspective of lymphadenectomy and an anatomic account of bladder lymphatic drainage are presented. The boundaries and technique of an extended lymphadenectomy are also highlighted. Autopsy and contemporary survival data are presented to suggest that a more extensive lymphadenectomy has both prognostic and therapeutic utility. Furthermore, the stage of the primary bladder tumor, total number of lymph nodes removed, and the lymph node tumor burden are shown to be important prognostic variables in patients undergoing cystectomy with pathologic evidence of lymph node metastasis. Radical cystectomy provides not only excellent local cancer control with low pelvic recurrence rates, but also the best long-term survival. Radical cystectomy with an appropriate extended lymphadenectomy, while surgically more challenging, does not significantly increase the morbidity or mortality of the procedure. Although the absolute limits of the lymph node dissection remain to be determined, there is an evolving body of data to support that an extended lymphadenectomy provides further diagnostic and therapeutic benefit.

## BACKGROUND

Bladder transitional cell carcinoma (TCC) makes up nearly 90% of all primary bladder tumors. In the U.S., it is the fourth most common cancer in men and the eighth most common cancer in women[1]. Approximately 70% of patients with bladder cancer initially present with superficial disease that has not invaded through the lamina propia. However, one-fourth of patients will either present with or subsequently develop muscle-invasive disease. If left untreated, over 85% of patients will die of the disease within 2 years of diagnosis[2]. Despite an early and aggressive approach toward high-grade, invasive bladder cancer[3], nearly 25% of patients demonstrate pathologic evidence of lymph node metastases at the time of cystectomy[4,5,6,7]. As a result, high-grade, muscle-invasive bladder cancer is typically regarded as a potentially lethal disease with high propensity for spread despite definitive therapy.

The rational for an extended lymphadenectomy in high-grade, invasive bladder cancer is based on the natural history of the disease process. Local invasion of bladder cancer can occur by either en bloc, lateral, or tentacular spread. The tumor progressively grows from its superficial origin in the mucosa to the muscularis propira, onto the periveiscal fat and contigous organs. At each site, tumor cells have access to blood vessels and lymphatics through which they may metastasize to regional lymph nodes or distant sites. This review will evaluate historical and contemporary aspects of the role of a lymphadenectomy in patients undergoing radical cystectomy for invasive TCC of the bladder.

## An Historical Perspective

The earliest surgical oncologists proposed the theoretical value of removing clinically palpable lymph nodes. In the late 18$^{th}$ century, Heister advocated axillary dissection for the treatment of breast cancer after observing the clinical course of patients with advanced disease[8]. Clinical observation in these patients suggested that the tumor spread in an orderly fashion from the primary site to the regional lymph nodes, and finally to distant locations. Based on these observations, the prevailing theory that developed (and was perpetuated until late in the 20$^{th}$ century) was that the lymph nodes served as physical barriers to tumor dissemination. Likewise, Halstead also introduced the concept of a regional lymph node dissection[9]. As a surgeon treating carcinoma of the breast, Halstead astutely noted an improved survival in locally advanced breast cancer following the application of a regional lymph node dissection with mastectomy. This concept has become one of the important principles of oncologic surgery; tumors tend to metastasize initially via the organ’s primary lymphatics to the regional lymph nodes. The removal of the primary tumor and regional lymph nodes provide important staging and possibly therapeutic benefits.

Our perception of advanced bladder cancer with regional lymphadenopathy has also changed appreciably over the past century. Early autopsy series of patients with bladder cancer in the 1930s reported a 17% incidence of isolated regional lymph node involvement[10]. Without fail, this was originally considered to be a fatal disease resistant to the therapeutic benefits of resection. However, this opinion was challenged both by Colston and Leadbetter[11] and subsequently by Jewett and Strong[12]. They demonstrated the often-limited metastatic spread of bladder cancer to only the regional lymph nodes and suggested that they were potentially amenable to surgical resection.

The survival benefits of a pelvic lymphadenectomy in patients with bladder cancer should be first credited to Drs. Kerr and Colby[13]. In 1950, they reported long-term survival in two node-positive patients undergoing radical cystectomy with a pelvic lymphadenectomy. In addition, the authors commented on their concerns of the high incidence of pelvic recurrence following a simple cystectomy. These observations prompted the routine application and inclusion of a lymphadenectomy with wide excision at the time of radical cystectomy. At the same time, Dr. Leadbetter reported his experience detailing his surgical approach and boundaries of an extended lymphadenectomy in patients with bladder cancer based on a better understanding of the lymphatic drainage of the bladder and prostate[14]. This included: (1) proximally, the distal aorta; (2) laterally, the genitofemoral nerve; (3) distally, the recurrent iliac vein (laterally) and the lymph node of Cloquet (medially). Although the specific boundaries or limits of a lymphadenectomy for bladder cancer continue to be debated today, the suggestion even in 1950 was that a more extended lymphadenectomy may be necessary to remove all potential lymph node metastases including the lymphatics at the level of the distal aorta and vena cava.

## Lymphatic Drainage of the Bladder

An anatomical understanding of the lymphatic drainage of the bladder is necessary when considering the specific sites of lymph node metastases, as well as attempting to best define the required boundaries of an appropriate lymphadenectomy. The primary contributions to the knowledge of bladder lymphatics have come from European sources and were well summarized by Leadbetter in 1950[14].

The lymphatic drainage from the bladder is accomplished by a system of lymphatic channels and lymph glands separated into 6 distinct areas: (1) the visceral lymphatic plexus within the bladder wall, initiating in the submucosa and extending into the muscular layer of the organ; (2) the intercalated lymph nodes, which are juxtavesical lymph nodes located within the perivesical fat arranged into anterior, lateral, and posterior groups; (3) pelvic collecting trunks, which are medial lymph nodes to the external iliac and hypogastric lymph nodes; (4) regional pelvic lymph nodes, which include the external iliac, hypogastric, and sacral lymph node groups; (5) lymphatic trunks leading from the regional pelvic lymph nodes to; (6) common iliac lymph nodes, those on the common iliac vessels, thought to be the secondary echelon of metastases, intermediate between the pelvic and the aortocaval lymph nodes[14].

Radical cystectomy series have confirmed that the two most common sites of lymph node involvement are the obturator and external iliac lymph nodes. Smith and Whitmore reported that these sites of nodal metastases were involved in 74 and 65%, respectively[15]. This study also demonstrated lymph node metastases in 19% of cases to the common iliac lymph node packet. This was one of the first anatomical lymph node mapping studies in patients undergoing radical cystectomy that suggested the importance of an extended lymph node dissection and removal of all potential lymph node metastases; including those along the common iliac vessels. The need to extend the lymph node dissection to a higher (more cephalad) level remains controversial. Leadbetter initially commented that it was not necessary to include the aortocaval lymph nodes since the surgical removal could not be done and should not be part of the cystectomy[14]. In fact, it has been shown that an extended lymphadenectomy, including removal of the lymphatic tissue distal to the inferior mesenteric artery, can be performed safely[4,5]. Furthermore, there is pathologic evidence to suggest that the lymph node region extending from the aortic bifurcation to the level of the inferior mesenteric artery may be a common site of nodal metastasis that can be effectively removed surgically[5].

Recently, the specific distribution of nodal metastases was prospectively evaluated in a multicenter study in which an extended lymphadenectomy was performed in all patients with bladder cancer[5]. This mapping study demonstrated that positive lymph nodes were found most commonly in the obturator spaces and adjacent to the iliac vessels. Interestingly, 16% of lymph node metastases also included nodes above the aortic bifurcation, while 8% of nodal metastases involved the presacral region. Among patients with nodal metastases located within the limits of a “standard” dissection (below the bifurcation of the common iliacs), a significant proportion of patients also had nodal involvement at the level of the common iliac vessels and above the aortic bifurcation; 57 and 31%, respectively. The authors noted that had the dissection been limited to the obturator spaces, 74% of all positive lymph nodes would have been left behind and nearly 7% of the patients in this cohort would have been misclassified as node negative[5]. The significance of an extended lymphadenectomy was also corroborated in a study that found 33% of patients with unexpected microscopic nodal involvement at the time of cystectomy to have metastases to the common iliac lymph nodes[16].

A stage-specific, lymph node metastasis mapping study was recently reported by Vazina and colleagues[6]. A total of 176 patients underwent an extended lymphadenectomy with radical cystectomy, in which 43 (24.4%) had pathologic lymph node involvement. Although the most common sites of nodal metastases were the external iliac and hypogastric/obturator regions, 5.1% had presacral nodal involvement and 9% had disease above the common iliac bifurcation. Importantly, 33% of patients with involvement of the common iliac lymph nodes also had involvement of the presacral region, supporting the importance of removing these nodes as well. Interestingly, a “skip metastasis” occurred in 1 patient with positive lymph nodes at or above the common iliac bifurcation without involvement of the more distal pelvic lymphatics[6]. Collectively, these studies support the application of a more extended lymphadenectomy with the cephalad extent of dissection that includes the distal para-aortic and paracaval lymph nodes, as well as removal of the presacral nodal packet.

The need for a bilateral lymph node dissection has also been questioned, particularly in patients with a unilateral bladder tumor[17,18]. In the mapping study by Leissner et al., bilateral lymph node metastases were commonly seen even if the primary cancer was limited to the right or left hemisphere of the bladder wall[5]. Mills and associates evaluated lymph node metastases in 83 patients with bladder cancer following radical cystectomy and found that 41% of patients with a unilateral bladder tumor had contralateral nodal involvement[19]. In a mapping study of 200 patients undergoing an extended lymphadenctomy, 24% of patients were found to have node-positive disease and 39% of these cases had bilateral involvement[20]. These data suggest that a bilateral lymphadenectomy is important to remove all potential sites for nodal metastases at the time of cystectomy. In addition, in view of the diversity and potential for bilateral spread of nodal metastases, the concept of a sentinel node lymphadenectomy may be difficult to apply and not readily transferable to bladder cancer treated with radical cystectomy.

## INCIDENCE OF LYMPH NODE METASTASIS FOLLOWING CYSTECTOMY

Similar to autopsy series, the incidence of lymph node metastases in patients undergoing radical cystectomy is approximately 26%[4,5,6,7,21,22]. In the 2815 patients undergoing radical cystectomy with lymphadenectomy in the referenced series, 723 patients (26%) were found to have lymph node metastases. In the largest reported cystectomy series of 1054 patients from the University of Southern California (USC), a total of 246 (24%) were found to have lymph node metastases[4]. The incidence of lymph node tumor involvement correlated with increasing tumor stage including: 5% with superficial, nonmuscle-invasive, primary bladder tumors (P0, Pa, Pis, P1); 18% with superficial, muscle-invasive tumors (P2a); 27% with deep, muscle-invasive tumors (P2b); 45% with extravesical tumors (P3); and 45% with P4 primary bladder tumors.

## SURGICAL BOUNDARIES AND TECHNIQUE OF THE LYMPHADENECTOMY

An “extended” lymphadenectomy must include all lymph nodes in the boundaries of the aortic bifurcation and common iliac vessels (proximally), the genitofemoral nerve (laterally), the circumflex iliac vein and lymph node of Cloquet (distally), the hypogastric vessels (posteriorly) including the obturator fossa, presciatic nodes bilaterally, and the presacral lymph nodes. As mentioned previously, an extended dissection may also extend superiorly to the level of the inferior mesenteric artery. A so-called “standard” lymphadenectomy is more limited with the cephalad extent generally beginning at the level of the common iliac bifurcation. The lateral and distal limits are similar to the extended dissection.

The meticulous extended lymphadenectomy is initiated at the superior portion of dissection, as all cephalad lymphatics are ligated with hemoclips to prevent lymphatic leak, while the caudal (specimen) side is ligated only when a blood vessel is encountered. Frequently, small anterior tributary veins originate from the vena cava just above the bifurcation, which should be clipped and divided. In men, the spermatic vessels are retracted laterally and spared. In women, the infundibulopelvic ligament along with the corresponding ovarian vessels have been previously ligated and divided at the pelvic brim. All fibroareolar and lymphatic tissues are dissected caudally off the aorta, vena cava, and common iliac vessels over the sacral promontory into the deep pelvis. The initial dissection along the common iliac vessels is performed over the arteries, skeletonizing them. Extra caution should be employed with patients who have undergone pelvic irradiation or when dissecting severely atherosclerotic vessels as this may inadvertently cause dislodgement and migration of plaque. As the common iliac veins are dissected medially, care is taken to control small arterial and venous branches coursing along the anterior surface of the sacrum. Electrocautery is helpful at this location, which allows the adherent fibroareolar tissue to be swept off the sacral promontory down into the deep pelvis, with the use of a small gauze sponge. Significant bleeding from these presacral vessels can occur if not properly controlled. Hemoclips are discouraged in this location as they can be easily dislodged from the anterior surface of the sacrum, resulting in troublesome bleeding. Once the proximal portion of the lymph node dissection is completed, a finger is passed from the proximal aspect of dissection under the pelvic peritoneum (anterior to the iliac vessels), distally toward the femoral canal. The opposite hand can be used to strip the peritoneum from the undersurface of the transversalis fascia and connect with the proximal dissection from above. This maneuver elevates the peritoneum and defines the lateral limit of peritoneum to be incised and removed with the specimen. In men, the peritoneum is divided medial to the spermatic vessels and lateral to the infundibulopelvic ligament in female patients. The only structure encountered is the vas deferens in the male or round ligament in females; these structures are clipped and divided.

A large, right-angled rake retractor (e.g., Israel) is used to elevate the lower abdominal wall, including the spermatic cord or remnant of the round ligament, to provide distal exposure in the area of the femoral canal. Tension on the retractor is directed vertically toward the ceiling, with care taken to avoid injury to the inferior epigastric vessels. This provides excellent exposure to the distal external iliac vessels. The distal limits of the dissection are then identified; the circumflex iliac vein crossing anterior to the external iliac artery distally, the genitofemoral nerve laterally, and Cooper's ligament medially. The lymphatics draining the ipsilateral leg, particularly medial to the external iliac vein, are carefully clipped and divided to prevent lymphatic leakage. This includes the lymph node of Cloquet, which represents the distal limit of the lymphatic dissection at this location. The distal external iliac artery and vein are then circumferentially dissected and skeletonized, with care taken to ligate an accessory obturator vein (present in 40% of patients) originating from the inferiomedial aspect of the external iliac vein. After completing the distal limits of dissection, the proximal and distal dissections are joined.

The proximal external iliac artery and vein are skeletonized circumferentially to the origin of the hypogastric artery. Care should be taken to clip and divide a commonly encountered vessel arising from the lateral aspect of the proximal external iliac vessels coursing to the psoas muscle. The external iliac vessels (artery and vein) are then retracted medially and the fascia overlying the psoas muscle is incised medial to the genitofemoral nerve. On the left side, branches of the genitofemoral nerve often pursue a more medial course and may be intimately related to the iliac vessels, in which case they are excised.

At this point, the lymphatic tissue surrounding the iliac vessels is composed of a medial and lateral component attached only at the base within the obturator fossa. The lateral lymphatic compartment (freed medially from the vessels and laterally from the psoas) is bluntly swept into the obturator fossa by retracting the iliac vessels medially and passing a small gauze sponge lateral to the vessels along the psoas and pelvic side wall. This sponge should be passed anterior and distal to the hypogastric vein, directed caudally into the obturator fossa. The external iliac vessels are then elevated and retracted laterally, and the gauze sponge carefully withdrawn from the obturator fossa with gentle traction using the left hand. This maneuver effectively sweeps all lymphatic tissue into the obturator fossa and facilitates identification of the obturator nerve deep to the external iliac vein.

The obturator nerve is best identified proximally and carefully dissected free from all lymphatics. The obturator nerve is then retracted laterally along with the iliac vessels. At this point, the obturator artery and vein should be carefully entrapped between the index finger (medial to the obturator nerve) laterally and the middle finger medially with the left hand. This isolates the obturator vessels exiting the obturator canal along the pelvic floor. These vessels are then carefully clipped and divided, ensuring that they stay medial to the obturator nerve. The obturator lymph node packet is then swept medially toward the side wall of the bladder, ligating small tributary vessels and lymphatics from the pelvic side wall.

After removing the cystectomy specimen, the presacral nodal tissue previously swept off the common iliac vessels and sacral promontory into the deep pelvis is collected and sent separately for pathological evaluation. Nodal tissue in the presciatic notch, anterior to the sciatic nerve, is also sent for histological analysis.

## MAXIMIZING THE NUMBER OF LYMPH NODES EVALUATED OR RETRIEVED

The number of lymph nodes assessed pathologically depends on several factors including: (1) the boundaries of the lymph node dissection (extended vs. standard or even more limited), (2) the pathologist's diligence in searching and preparing the lymph nodes for histopathologic evaluation, and (3) how the specimen is actually submitted for pathologic evaluation. These factors may collectively contribute to the determination of the actual number of lymph nodes retrieved and the exact incidence and extent of lymph node tumor involvement.

Diligent pathologic evaluation is essential for the identification of the total number of nodes removed and the amount of nodal metastases. In general, most lymph nodes are identified visually and by palpation, without the need of clearing techniques or solvents. With this technique, in a large group of 244 patients with lymph node–positive disease, undergoing an en bloc radical cystectomy and extended lymphadenectomy, a median number of 30 lymph nodes were removed and evaluated[23]. It has recently been suggested that in order to facilitate nodal evaluation, the surgeon should submit separate nodal packets intraoperatively[24]. Simply converting from an en bloc technique to submission of 6 separate lymph node packets (while maintaining the limits of dissection), the mean number of lymph nodes removed increased by more than threefold[16]. We have adopted a similar approach at USC with submission of 12 individual lymph node packets. This modification has significantly increased the median number of lymph nodes removed/evaluated from 30 to 56 (unpublished data).

The absolute limits of the lymph node dissection may be the most important factor and have the greatest impact on the number of lymph nodes removed during cystectomy. In two large cystectomy series, in which an extended lymphadenectomy was performed, the median number of lymph nodes removed ranged from 30–43[5,23]. Extending the boundaries of the lymph node dissection, Poulsen et al. reported an increase in the average number of lymph nodes removed from 14 in a standard dissection to a total of 25 when the dissection was carried up to the bifurcation of the aorta[21]. Others have confirmed these findings, reporting a significantly greater number of nodes removed with an extended dissection compared to a more standard dissection[24,25].

It is clear that the influence of the surgeon and pathologist are both important factors in determining the lymph node count and involvement of tumor. Although the exact number of nodes that should be removed at the time of cystectomy is unknown, it appears that extending the limits of the dissection and submitting the lymph nodes in packets increases the number of lymph nodes retrieved and evaluated. Fat-clearing, immunohistochemical, and molecular techniques may increase the nodal counts, but these specialized methods are more expensive, time consuming, and may not necessarily provide any additional prognostic information, particularly if an extended lymphadenectomy is performed. In fact, a recent report evaluated various factors that contribute to the variability in the number of reported lymph nodes removed at cystectomy; only the extent of the lymph node dissection was found to significantly influence the nodal yield[16].

## WHAT IS THE REQUIRED NUMBER OF LYMPH NODES THAT MUST BE REMOVED? THE IMPACT OF THE SURGICAL PROCEDURE

There is a growing body of evidence in bladder cancer and in other malignancies requiring surgical excision to suggest that a minimum number of lymph nodes should be removed and evaluated. The dedication and technical commitment of the surgeon to a properly performed cystectomy with an adequate lymphadenectomy is important to the success and clinical outcomes in patients with high-grade bladder cancer. The importance of surgical technique is well illustrated in the role this played in a recently reported, randomized, multi-institutional cooperative group trial[26]. In this prospective study, 270 patients underwent cystectomy with half of the patients receiving neoadjuvant chemotherapy. In a separate analysis of this trial, various surgical factors were subsequently analyzed[27]. In these 270 patients, 24 had no lymph node dissection, 98 had a limited dissection of the obturator lymph nodes only, and 146 patients had a so-called standard (not extended) pelvic lymph node dissection. The 5-year survival rates for these groups were 33, 46, and 60%, respectively. The median number of lymph nodes removed for the entire cohort was 10. As expected, the survival rate for patients with less than 10 lymph nodes removed was significantly lower compared to patients with more than 10 lymph nodes removed; 44 vs. 61%, respectively. In a multivariate analysis, the extent of the lymph node dissection, number of lymph nodes removed, and the number of cases performed by the individual surgeon were the most significant factors influencing survival in patients undergoing cystectomy for bladder cancer. Although this well-publicized study was not intended to analyze the surgical approach and/or technical differences in the treatment of bladder cancer, it is emphasized that it was the surgical factors, not the neoadjuvant chemotherapy, that were most critical as predictors in the outcomes of these patients[27].

Due to varied surgical and technical approaches in the treatment of bladder cancer among urologists, guidelines in the surgical approach and clinical outcomes of patients with TCC undergoing cystectomy have recently been proposed. A multicentered report from the Bladder Cancer Collaborative Group evaluated and suggested some surgical standards for radical cystectomy[28]. A total of 16 experienced surgeons form 4 academic institutions contributed 1091 cystectomy patients over a 3-year period of time. The authors concluded that at least 10 yearly cystectomies are required to maintain proficiency. At least 10–14 lymph nodes should be retrieved, with a margin-positive rate of fewer than 10% of all cases (less than 15% for bulky tumors and less than 20% for salvage cases) performed. It is noteworthy to mention that the authors also found that a complete/standard lymphadenectomy correlated with fewer positive margins and increased node counts in patients with positive and negative nodal disease[28]. This, too, argues for a more extensive lymphadenectomy in patients undergoing radical cystectomy for bladder cancer.

## MORBIDITY AND MORTALITY OF LYMPHADENECTOMY

Understanding that a lymph node dissection is important in the management of patients undergoing radical cystectomy for bladder cancer, coupled with the fact that a more extensive lymphadenectomy may provide more accurate pathologic staging and survival benefits, one must carefully evaluate the risks associated with an extended lymph node dissection. This is an important issue particularly in bladder cancer patients who tend to be elderly with associated comorbidities.

A recent study questioned whether an extended lymphadenectomy would increase morbidity in patients undergoing radical cystectomy[29]. A total of 46 patients undergoing an extended lymphadenectomy (cephalad dissection at the level of the inferior mesenteric artery) was compared to 46 patients undergoing a more “standard” dissection, with the cephalad extent at the region of the common iliac artery bifurcation. Patients were well matched with regard to associated comorbidities and American Society of Anesthesiologist (ASA) grade. Overall, a total of 30% of patients were found to have lymph node metastases. Although the extended lymphadenectomy increased the operative duration by 63 min, there was no significant difference in perioperative mortality, early complications, or the need for blood transfusions between the two groups. The authors concluded that despite prolonging the operation, an extended lymphadenectomy does not apparently result in an increased complication rate during or after (within 30 days following) surgery. Likewise, in the USC series of 1054 patients, a subgroup analysis of patients with lymph node–positive disease carried an operative mortality of 1%, with an early complication rate of 27%[23]. No differences were noted in operative mortality and morbidity when comparing this pathologic group to those patients without lymph node tumor metastases. The findings of other studies have collectively shown that the morbidity associated with an extended lymphadenectomy is low and comparable to a more limited node dissection[21,30].

Although an extended lymph node dissection on average may require 60 min longer to perform than a more limited lymphadenectomy, it should be noted that during the immediate postoperative period, no significant adverse effects are directly related to an extended lymphadenectomy[5].

## PROGNOSTIC FACTORS IN PATIENTS WITH LYMPH NODE METASTASES FOLLOWING RADICAL CYSTECTOMY

Patients with lymph node metastases are at higher risk for tumor recurrence and progression compared to other pathologic subgroups (organ-confined and extravesical, lymph node–negative tumors)[4,21,22]. However, nearly a third of patients will demonstrate long-term survival following radical cystectomy[4,23]. To provide risk stratification and better direct the need for adjuvant treatment therapies, various prognostic factors have been identified in patients with lymph node metastases following radical cystectomy. These prognostic factors include the extent of the primary bladder tumor (p-stage), the total number of lymph nodes involved with tumor (tumor burden), the extent of lymphadenectomy (number of lymph nodes removed), and lymph node density.

### Pathological Stage

The pathological stage or extent of the primary bladder tumor remains one of the strongest prognostic indicators of patients with node-positive disease following radical cystectomy[4,7,22,23]. Patients with organ-confined (P0-P3a), node-positive disease have a 46–58% probability of surviving 5 years, compared to a 22–30% 5-year survival in patients with extravesical, lymph node–positive disease[22,23]. Furthermore, in a multivariate analysis, the extent of the primary bladder tumor remains a significant and independent prognostic factor in patients with lymph node–positive tumors[22].

### Tumor Burden

The number of positive lymph nodes, or number of lymph nodes involved with tumor (tumor burden), is recognized as an important prognostic factor in patients with bladder cancer following radical cystectomy[4,7,19,23,30,31]. As expected, survival and recurrence are inversely related to an increasing tumor burden[15]. In the largest reported series of 244 lymph node–positive patients with median follow-up of 10 years, the number of lymph nodes involved with tumor was a significant and independent prognostic factor regarding survival in patients following cystectomy[23]. A similar report on 132 patients with nodal metastases found that patients with 5 positive lymph nodes or less had a significantly better recurrence-free and overall survival compared to those with 6 or more lymph nodes involved with tumor[32]. Likewise, Herr and associates found that survival was significantly improved for patients with node-positive disease if the patients had 4 or fewer positive lymph nodes, compared to those with greater than 4 positive lymph nodes (37 vs. 13%, respectively)[33]. In this node-positive group of patients, if more than 11 lymph nodes were removed (total), an improved survival with better local pelvic control of the tumor was observed. These data underscore the importance of a more extended lymphadenectomy in patients with node-positive bladder cancer. Interestingly, in the subgroup of lymph node–negative patients, survival was also directly proportional to the number of lymph nodes removed. The authors appropriately comment that the more lymph nodes identified may reflect a more complete radical cystectomy and lymphadenectomy in both lymph node–positive and –negative patients.

### Extent of Lymphadenectomy

Although the absolute limits or extent of the lymph node dissection have not been precisely defined, there is a growing body of data to suggest that a minimum number of lymph nodes should be removed and pathologically evaluated during radical cystectomy[23,28,30,33]. The number of lymph nodes removed not only suggests the completeness of the lymph node dissection, but also appears to have prognostic significance in both lymph node–positive and –negative patients with bladder cancer following radical cystectomy

As aforementioned, Poulsen et al. demonstrated that extending the limits of the node dissection from the bifurcation of the common iliac vessels up to the level of the aortic bifurcation increased the median number of lymph nodes removed from 14–25[21]. It has been shown that survival for both lymph node–negative and –positive patients is improved, with a reduced local recurrence rate, when a greater number of lymph nodes are removed[30]. In Leissner's study, if more than 16 lymph nodes were removed, the 5-year, recurrence-free survival increased from 63–85% in organ-confined tumors, from 40–55% in pT3 tumors, and from 25–53% in patients with at most 5 lymph node metastases. Furthermore, if at least 20 lymph nodes were removed, approximately 80% of lymph node–positive patients would be identified, suggesting that this would be a reasonable number of lymph nodes to be removed and evaluated at cystectomy.

Similarly, extending the limits of the pelvic lymph node dissection provides a benefit in the subset of patients with organ-confined, lymph node–negative bladder tumors[21]. The 5-year recurrence-free survival with organ-confined, node-negative tumors was 85% with an extended dissection compared to 64% with similar pathology undergoing a more limited dissection. Furthermore, an extended dissection reduced the pelvic and distant metastases rate in these patients. Additional confirmation comes from an analysis of over 20,000 bladder cancer patients (1923 patients undergoing cystectomy) included in the SEER cancer registry[34]. In this large cohort, it was determined that the risk of death was significantly higher in patients with less than 4 lymph nodes removed at cystectomy, independent of stage and lymph node–positive disease. In this study, the most important survival factor in patients undergoing cystectomy, effectively controlling for age, tumor stage, histology, chemotherapy and radiation therapy, was the removal of 10–14 lymph nodes at the time of surgery[34].

## Lymph Node Density

Lymph node density simultaneously accounts for the extent of lymph node dissection (number of lymph nodes removed) and tumor burden (number of positive lymph nodes) following radical cystectomy in patients with lymph node–positive disease. Lymph node density is defined as the number of lymph nodes involved with tumor divided by the total number of lymph nodes removed. If tumor burden and the extent of the lymphadenectomy are important variables in patients with node-positive bladder cancer, it is logical that lymph node density should also be prognostic. Lymph node density better stratifies lymph node–positive patients into various risk groups, which may be useful in future staging systems.

In the USC group of 244 lymph node–positive patients, lymph node density was found to be a significant and independent prognostic factor. Patients with a lymph node density of 20% or less, demonstrated a 43% 10-year recurrence-free survival compared to only a 17% survival at 10 years, when the lymph node density was greater than 20%[23].

Herr recently published his findings regarding this concept of lymph node density, described as ratio-based lymph node staging[35]. In 162 patients with lymph node–positive disease, this ratio system better defined the surgical outcomes in these patients. The 5-year survival in patients with node-positive disease and a lymph node density of less than 20% was 64%, significantly higher than the 8% 5-year survival for the same pathologic group of patients with a lymph node density greater than 20%[35]. Similarly, the proportion of positive lymph nodes to excised lymph nodes (lymph node density) for metastatic bladder cancer correlated with the risk of death from bladder cancer in the SEER registry of patients undergoing radical cystectomy[34].

## Extranodal Growth

Extracapsular extension of lymph node metastases, which is defined as perforation of the capsule by tumor tissue with extranodal growth, has recently been shown to double the risk of recurrence when compared to intranodal confined lymph node metastasis[36]. The study from Studer's group in Bern, Switzerland found that 58% of patients with node-positive disease exhibited evidence of extranodal growth, which histopathologically must be differentiated from tumor deposits in the pericapsular lymphatics. In a multivariate analysis including tumor stage, number of lymph nodes involved, and lymph node density, extracapsular extension of lymph node metastases was the strongest negative predictor of recurrence-free survival[36].

## CONCLUSIONS

Radical cystectomy with bilateral pelvic iliac lymphadenectomy is a standard treatment for high-grade, invasive bladder cancer. Cystectomy arguably provides the best survival outcomes and the lowest local recurrence rates. Although the extent or absolute limits of the lymph node dissection is unknown and remains to be better defined, there is an ever-growing body of data to support a more extended lymphadenectomy at the time of cystectomy in all patients that are appropriate surgical candidates. It appears that an extended lymph node dissection should include the distal para-arotic and paracaval lymph nodes, as well as the presacral nodes (known anatomical sites of lymph node drainage from the bladder, as well as potential sites of lymph node metastases in patients with bladder cancer). It appears that an extended dissection may provide a survival advantage in both node-positive and -negative tumors without significantly increasing the morbidity or mortality of the surgery. The extent of the primary bladder tumor (p-stage), number of lymph nodes removed, and the lymph node tumor burden are all important prognostic variables in patients undergoing cystectomy with pathologic evidence of lymph node metastases. Lymph node density may become an even more useful prognostic variable in these high-risk, node-positive patients with bladder cancer. This concept simultaneously incorporates both the lymph node tumor burden (number of lymph nodes involved) and the number of lymph nodes removed (extent of the lymphadenectomy), which may improve stratification of lymph node–positive patients following radical cystectomy. This notion may also be useful in future staging systems. Furthermore, adjuvant therapies and clinical trials should consider applying these concepts as it may help reduce bias and incorporate the extent of the lymphadenectomy, which is currently not standardized.
